# Evidence of sexual dimorphism in skeletal morphology of a gonochoric reef coral

**DOI:** 10.1098/rsos.171843

**Published:** 2018-05-23

**Authors:** P. C. González-Espinosa, D. A. Paz-García, H. Reyes-Bonilla, R. A. Cabral-Tena, E. F. Balart

**Affiliations:** 1 Laboratorio de Necton y Ecología de Arrecifes, Centro de Investigaciones Biológicas del Noroeste (CIBNOR), La Paz, Baja California Sur, México; 2 Laboratorio de Sistemas Arrecifales, Universidad Autónoma de Baja California Sur (UABCS), La Paz, Baja California Sur, México

**Keywords:** sexual dimorphism, corallite, morphology, reef-building coral, *Porites panamensis*

## Abstract

In the emerald coral *Porites panamensis*, the rates of elongation and calcification of colonies are higher in males than in females, probably because of the higher energetic demands of the latter in order to cope with the development of the large planulae produced throughout the year. This differing energetic demand could also be reflected in the sexual dimorphism of the calyces; hence, to test this hypothesis, 11 morphological traits of the corallite were assessed from 63 colonies that were collected in the southern Gulf of California, Mexico. Three traits showed statistical differences between sexes, enabling accurate distinction of males from females. Our results confirm for the first time the existence of external sexual dimorphism in a reef-building coral, opening the possibility that sex-related morphological differences may occur generally in gonochoric scleractinians. These findings can be very useful for the correct classification and characterization of recent and fossil records, helping to improve the historical and evolutive understanding of reef-building corals facing threats under environmental changes.

## 1. Introduction

Sexual dimorphism is widely known in the animal kingdom. There are examples in marine invertebrates, such as molluscs and echinoderms [1–3], but little is known about this condition in scleractinian corals. Recent studies in the gonochoric corals, *Porites panamensis* [4], *Porites lobata* [5], *Montastraea cavernosa* [6] and *Siderastrea siderea* [7], found that growth rates (i.e. extension, calcification and density) are significantly higher in males than females. Additionally, environmental conditions and coral growth affect skeletal isotopic signals differently in each sex [8]*.* This sex effect on carbonate accretion and allocation in coral skeleton allowed us to hypothesize a sexual dimorphism in skeletal morphology in a gonochoric coral *P. panamensis.*

The coral *P. panamensis* is endemic to the eastern tropical Pacific and has a wide latitudinal distribution from the upper Gulf of California to Colombia [9]. This coral copes with a wide range of environmental conditions, including low temperature, low pH and high turbidity levels that are often considered unsuitable for coral development [10]. Here, we report the first evidence of sexual dimorphism in corallite morphology in a scleractinian coral. The study was based on the measurement and comparison of morphological traits of corallites in males and females of the coral *P. panamensis*, collected at Bahía de La Paz in the southern Gulf of California.

## 2. Material and methods

### 2.1. Sample collection

**Figure 1.**
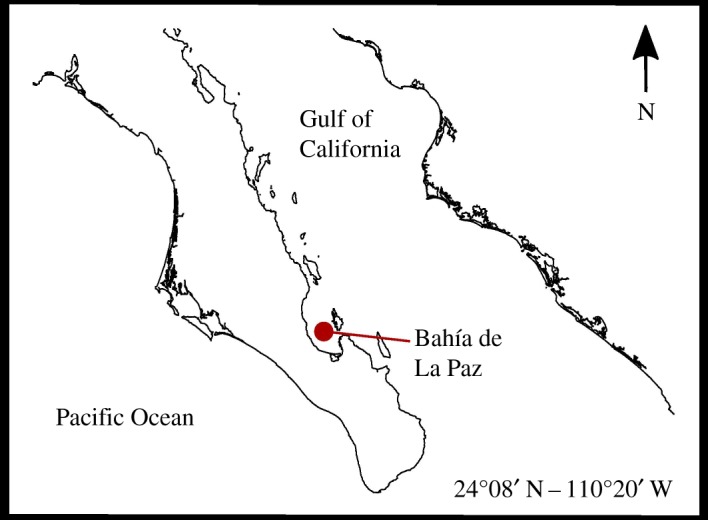
Location of study site in the Gulf of California; Bahía de La Paz (BLP).

The samples were collected in Punta Gaviotas reef, Bahía de La Paz in the southern Gulf of California, México (24°08′ N, −110°20′ W; figure 1). In total, 63 coral colonies were collected using a hammer and chisel to remove the fragments from colonies. All colonies were sampled in shallow water 3–7 m in depth during the reproductive period (March to July), and under the approval of the Mexican Secretariat of Agriculture, Livestock, Rural Development, Fisheries and Food (SAGARPA).

### 2.2. Sex identification

Two fragments for each colony were collected; one was used for morphological analysis, and the second fixed in Davison solution for sex identification with subsequent histological processing (see [4] for method details). Female sex determination was made by microscopic differentiation of oocytes (regardless of their stage of development) or if any planulae were observed, and colonies were considered male if any spermatocytes were observed on the slide.

### 2.3. Morphometric analysis and corallite traits

**Figure 2.**
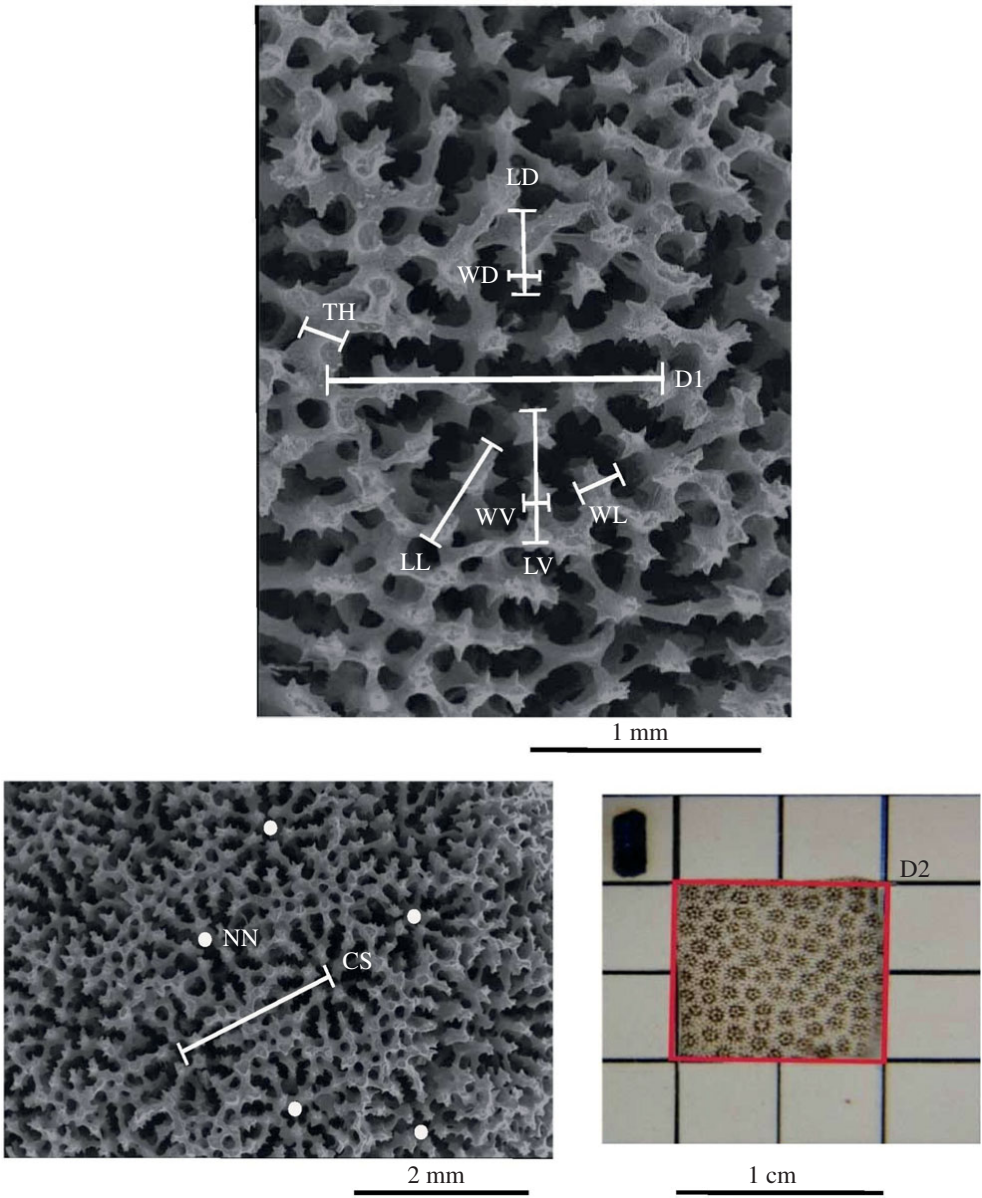
Corallite traits of *P. panamensis*: calical spacing (CS), corallite diameter (D1), corallite density (D2), length of dorsal septum (LD), length of lateral septum (LL), length of ventral septum (LV), number of neighbouring corallites (NN), wall thickness (TH), width of dorsal septum (WD), width of lateral septum (WL), width of ventral septum (WV).

To test morphological differences and characterize sexual dimorphism in corallite morphology, 11 traits were measured in 10 corallites selected haphazardly per fragment (colony); each trait measurement was then averaged to get a single mean value per colony and per trait (figure 2). A literature review was conducted and the traits were selected according to their usefulness to identify *Porites* species [11–13] (table 1). Pictures of each corallite at 2× and 4× objectives were taken using a Moticam 2500 digital camera (5.0 MP resolution) attached to a stereoscopic microscope OLYMPUS SZ40. Morphological traits were measured from corallite photographs using the ImageJ 1.34 software. Images were calibrated with a grid of known dimensions.

**Table 1.** List and description of all morphologic traits measured on *Porites panamensis* corallites.

trait	code	description
1. calical spacing	CS	average of the longest and shortest distance between centres of neighbouring corallites
2. corallite diameter	D1	linear measure of the corallite diameter
3. corallite density	D2	number of corallites per square centimetre
4. length of dorsal septum	LD	linear measure of the distance from the calical wall to the end of the dorsal septum
5. length of lateral septum	LL	linear measure of the distance from the corallite wall to the end of the lateral septum
6. length of the ventral septum	LV	linear measure of the distance from the corallite wall to the end of the ventral septum
7. number of neighbouring corallites	NC	count of the number of adjacent corallites
8. wall thickness	TH	linear measure between thecal margins of nearest neighbouring corallites
9. width of dorsal septum	WD	width measured at the midpoint of the dorsal septum
10. width of lateral septum	WL	width measured at the midpoint of the lateral septum
11. width of the ventral septum	WV	width measured at the midpoint of the ventral septum

### 2.4. Statistical analysis

Morphometric studies in *Porites* and other scleractinian corals have been carried out using colony means of traits to characterize the morphological variation of the coral colony due to high plasticity and corallite variation that these organisms show [14–17]. The statistical analyses were performed using colony means. To assess how accurately males could be distinguished from females, we compared male and female corallite's means of traits per colony through a discriminant analysis.

A Welch's *t*-test was used to assess statistical differences in individual traits between sexes. If the data for a trait were not normally distributed, a Mann–Whitney–Wilcoxon Test was applied.

## 3. Results

**Figure 3.**
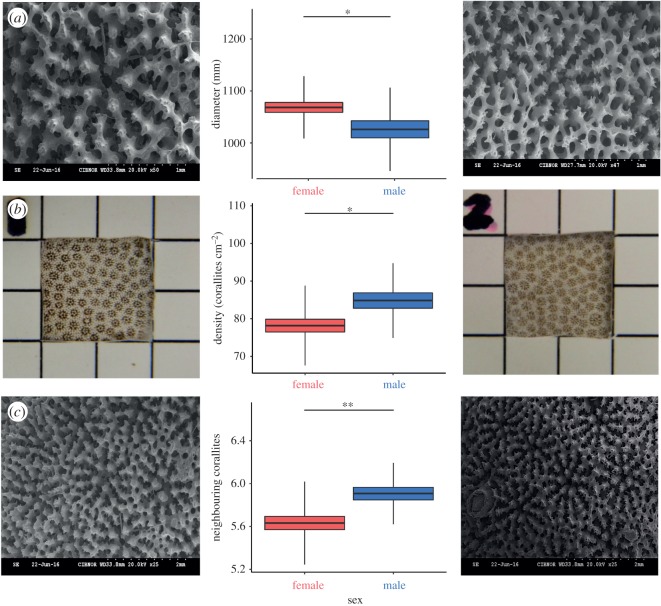
Significant skeletal traits of female (left/red) and male (right/blue) colonies of *P. panamensis* of Bahía de La Paz. Dark line in the box: mean, box: s.e., whisker: s.d. Significance codes: ‘**’ 0.01, ‘*’ 0.05, NS non-significant. (*a*) Diameter, (*b*) density per square centimetre, (*c*) number of neighbouring corallites.

A consistent pattern of sexual dimorphism was found in the scleractinian coral *Porites panamensis* in three out of eleven morphological traits analysed (figure 3). Corallite diameter was significantly wider (table 2) in the females than males (*t*_61_ = 2.38, *p*-value = 0.020), while males had a higher corallite density per square centimetre (*t*_61_ = −2.48, *p*-value = 0.016) and number of neighbouring corallites (MW = 322.5, *p*-value = 0.005).

**Table 2.** Descriptive statistics chart for female and male traits measurements. Italics indicate significant traits.

	CS	*D1*	*D2*	LD	LL	LV	*NC*	TH	WD	WL	WV
females											
min	959.91	943.17	54.33	180.63	286.15	319.48	5.00	56.62	75.04	84.02	102.29
max	1345.53	1180.07	101.00	314.25	476.71	495.08	7.00	184.94	168.62	164.38	169.44
mean	1099.58	1068.40	78.16	239.33	398.65	411.64	5.72	121.67	126.11	138.26	133.04
s.e.	12.56	9.56	1.69	5.21	6.38	5.73	0.08	5.22	3.37	2.32	2.22
s.d.	78.45	59.71	10.57	32.51	39.86	35.80	0.51	32.60	21.02	14.47	13.89
males											
min	950.75	792.72	67.50	159.86	319.32	311.36	6.00	82.83	82.46	109.16	108.46
max	1231.67	1221.44	106.00	321.59	441.45	474.86	7.00	185.78	184.36	179.80	165.13
mean	1086.84	1026.27	84.83	225.93	392.15	398.55	6.04	127.57	135.34	145.01	140.15
s.e.	13.58	16.34	2.02	8.41	6.75	7.26	0.04	6.01	4.02	3.50	2.81
s.d.	66.54	80.07	9.90	41.19	33.06	35.54	0.20	29.46	19.70	17.14	13.77

The discriminant analysis confirmed the sexual dimorphism in the coral *P. panamensis* (Wilks' lambda 0.635 approx., *F*_11,51_ = 2.665, *p* = 0.008), with 82% and 66% of females and males correctly classified, respectively. The most important morphological traits in the discriminant analysis to detect sexual dimorphism were the corallite diameter and neighbouring corallites (table 3).

**Table 3.** Standardized coefficients for canonical variables. The highest value is indicated in italic.

	Root 1
CS	0.345
D1	−*0*.*597*
D2	0.238
LD	−0.266
LL	−0.077
LV	−0.333
NC	0.450
TW	0.289
WD	0.372
WL	0.143
WV	0.389

## 4. Discussion

Sexual dimorphism in morphological traits was found in this study. Evidence of different growth rates and the hypothesis of different energetic demands between male and female colonies in gonochoric species of scleractinian corals [4–7] led us to test and confirm the sexual dimorphism in morphological traits at corallite level. Considering this, density (corallites cm^−2^), corallite diameter and neighbouring corallites can be used to identify sexes in *P. panamensis* colonies. This information can also be very useful, for example, to make comparisons using fossil material (the species has existed in the eastern Pacific since the Pliocene [13]), and to revise historical sex proportions in more recent time periods using museum specimens.

The sexual dimorphism found here for particular traits might be much influenced by reproductive characteristics. Most brooder corals develop larvae 2 mm in length [18] and *P. panamensis* is no exception as the planulae are between 210 and 350 µm in length; the polyps host up to three larvae growing simultaneously in their mesenteries, and reproduce almost all year long [19,20]. We suggest that the need to harbour fully developed larvae has been the selective pressure that led this coral to have a sizeable internal space, and secondarily, to diminish the density of polyps per square centimetre and the number of neighbouring corallites.

An interesting note to mention about different analysed samples from northern sites of the Gulf of California (Bahía de Los Angeles and Bahía Concepción) is that sexual dimorphism not only seems to vanish but also corallites appear to be bigger in both sexes (P.C.G.-E., D.A.P.-G., H.R.-B., R.A.C.-T. & E.F.B. 2017, personal observation). Within the Gulf of California environmental variables vary along a latitudinal gradient, displaying a higher seasonality and variability in the northern section of the gulf, and thus more stressful conditions [21,22]. Each region has distinct oceanographic characteristics; Bahía de Los Ángeles is located at high latitude (29° N) where the sea surface temperature (SST) varies from 16° to 29°C during the year [23], and the area presents permanent high nutrient concentration and productivity because of local upwelling and tidal mixing [24]. Bahía Concepción (26° N) and La Paz (24° N), which are closer to the entrance to the Gulf of California, show prevailing oligotrophic waters [25]. The possible reasons why sexual dimorphism is not evident in the northern sites of the Gulf of California (i) could be related to the presence of cryptic species as has been seen with dwellers of marginal environments at similar latitudes in the northwest Pacific [26], where the potential for population genetics studies would help to support these findings, or (ii) could be linked to the feeding mode. In the latter case, it is possible that polyps and calyces of both sexes in the north must to be larger because they receive relatively low light irradiance and switch to a greater influence of heterotrophism [27] or need a larger surface to accommodate a greater number of zooxanthellae. On the other hand, in the south, where solar radiation is higher and the turbidity is less, *P. panamensis* behaves autotrophically [28], and sex differences in corallites' skeletal morphology seem to become evident.

In conclusion, sexual dimorphism is detectable in three morphological traits of the reef-building coral *P. panamensis* (corallite diameter, the number of neighbouring corallites and the density of corallites per area unit). These morphological differences could be linked to the fact that female polyps harbour large gametes and planula larvae for many months during the year. In addition, following studies should be directed to address the environmental effect in the morphology of the corallites, as the diameters are apparently larger in northern areas of the Gulf of California, possibly as a response to a combination of low irradiance and high Chl-a concentration (P.C.G.-E., D.A.P.-G., H.R.-B., R.A.C.-T. & E.F.B. 2017, personal observation) that enable heterophagy or to accommodate a greater number of zooxanthellae. Finally, it is important to recall that according to the findings of this study, sex identification of collected material lacking tissue would enable more precise analyses of coral growth parameters and their associated palaeoclimatic reconstructions (e.g. use of stable isotopes in coral skeletons) by avoiding the implicit data variability of non-sex-separated samples as mentioned in previous studies [4–7].

## Data accessibility

The datasets supporting this article have been uploaded in the Dryad data repository (https://doi.org/10.5061/dryad.5d6sp).
